# CT Imaging Predictors of Early Neurologic Deterioration and 90-Day Outcomes in Thrombolysed Acute Ischemic Stroke: A Retrospective Single-Center Study

**DOI:** 10.2174/0115734056417932260627205752

**Published:** 2026-07-07

**Authors:** Yunjian Li, Xianxian Zhang, Tingting Wang, Suyan Han, Chaohui Zheng, Yan Li, Liping Zhang, Senhao Zhang, Xueyang Wang

**Affiliations:** 1 Department of Radiology, The Affiliated Hospital 6 of Nantong University, Yancheng Third People’s Hospital, Yancheng, China; 2 Department of Neurology, The Affiliated Hospital 6 of Nantong University, Yancheng Third People’s Hospital, Yancheng, China; 3 Department of Radiology, Yancheng Traditional Chinese Medicine Hospital Affiliated to Nanjing University of Chinese Medicine, Yancheng, China; 4 Department of Radiology, Yancheng Traditional Chinese Medicine Hospital, Yancheng, China; 5 Department of Radiology and Nuclear Medicine, Xuanwu Hospital, Capital Medical University, Beijing, China

**Keywords:** Stroke, Large vessel stenosis, Prognosis, Infarction, rLMC score, Clinical decision-making

## Abstract

**Objectives:**

This study aimed to evaluate the association between multimodal CT imaging parameters and Early Neurologic Deterioration (END) and 90-day functional outcomes in patients with acute anterior-circulation ischemic stroke treated with intravenous thrombolysis.

**Methods:**

In this retrospective single-center study, patients with anterior-circulation ischemic stroke who underwent multimodal CT imaging, including non-contrast CT, CT angiography, and CT perfusion prior to thrombolysis, were analyzed. Imaging parameters, including ASPECTS, regional leptomeningeal collateral (rLMC) score, infarct core, perfusion lesion, and ischemic penumbra volumes, were quantified using RAPID software. Multivariable logistic regression models and receiver operating characteristic (ROC) curve analyses were applied to identify independent predictors of END and of long-term functional outcome, as measured by the 90-day modified Rankin Scale.

**Results:**

A total of 131 patients were included, of whom 16% developed END. Lower rLMC scores and larger infarct core, perfusion lesion, and ischemic penumbra volumes were significantly associated with a higher risk of END and unfavorable 90-day outcomes. Perfusion lesion volume showed the strongest discrimination for predicting END, while the rLMC score demonstrated the highest predictive value for a favorable long-term functional outcome. ROC-derived thresholds exhibited only moderate accuracy and are considered exploratory.

**Discussion:**

These findings highlight the prognostic relevance of collateral status and CT-derived perfusion parameters in identifying patients at higher risk of neurologic worsening and long-term disability after thrombolysis.

**Conclusion:**

Multimodal CT imaging parameters, particularly rLMC score and perfusion-based tissue volumes, are associated with END and 90-day outcomes following thrombolysis. Further multicenter validation is necessary before clinical implementation.

## 1 INTRODUCTION

Acute ischemic stroke constitutes a primary cause of morbidity and mortality on a global scale, placing a considerable burden on healthcare systems and society [1, 2]. AIS improves functional outcomes and reduces disability [3, 4]. However, early neurologic deterioration following thrombolysis can adversely affect patient outcomes and remains a major clinical challenge [5]. Identifying clinical, laboratory, and radiological parameters related to long-term functional outcomes in patients with thrombolysis-treated symptomatic acute anterior circulation ischemic stroke is critical for risk stratification, therapeutic decision-making, and patient care optimization.

Computed tomography is commonly used as an imaging technique in the evaluation of AIS patients, given its availability, speed, and relatively lower cost. [6]. In recent years, CT imaging combined with advanced computational and deep learning techniques has emerged as a promising approach for early prediction of stroke outcomes. Recent studies using CT angiography demonstrate that cerebral collateral grading is an independent predictor of neurological and functional outcomes in acute anterior circulation ischemic stroke patients receiving thrombolytic therapy, highlighting the prognostic value of CTA in treatment stratification [7, 8]. Numerous studies have shown that various CT imaging parameters, such as infarct core volume, ischemic penumbra volume, and collateral circulation, can be used to evaluate clinical outcomes in AIS patients [9, 10]. Nonetheless, the correlations between some imaging-quantified parameters and the risks of END and LFO need to be elucidated. Neural networks are computational models inspired by the human brain, consisting of interconnected layers of nodes that process data. They learn patterns and relationships from input data to perform tasks like classification and prediction. The growing body of evidence supporting the use of endovascular thrombectomy in AIS patients presenting with major artery occlusions has highlighted the importance of understanding the role of CT imaging features in gauging their likelihood of responsiveness to thrombolysis and predicting their long-term outcomes [11, 12]. Recent studies demonstrate that CT-based collateral assessment is closely linked to outcomes in acute ischemic stroke treated with thrombolysis and endovascular thrombectomy, primarily influencing early infarct growth dynamics. However, the role of other CT imaging parameters, such as perfusion lesion volume, [13-15] has not been fully investigated. The relationship between Early Neurological Deterioration (END) and Long-term Functional Outcome (LFO) with radiological variables, particularly in patients receiving thrombolysis for symptomatic acute anterior circulation ischemic stroke, also remains insufficiently explored.

## 2 METHODS

### 2.1 Clinical Evaluation and Patient Selection

The research has been evaluated by our Medical Ethics Committee at Affiliated Hospital 6, Nantong University, and Yancheng Third People's Hospital, China (No. 2023-11). This study is based on records from prospective stroke registries maintained by our Stroke Centre, which included patients undergoing thrombolysis between March 2018 and February 2020. Baseline characteristics and 90-day outcomes of cases treated with thrombolysis within 24 hours of the last time the patient was recorded as having a good clinical condition were collected retrospectively. The reference to a 24-hour time window reflects the period for diagnostic evaluation and patient identification, rather than the administration of intravenous thrombolysis beyond guideline-recommended time limits. All patients received intravenous thrombolysis according to standard indications. Patients with wake-up stroke or unknown time of symptom onset were treated only after imaging-based selection using multimodal CT, including the absence of extensive early ischemic changes on non-contrast CT (NCCT) and the presence of salvageable tissue on CT perfusion imaging. In addition to conventional clinical evaluation, CT imaging data were further analyzed using quantitative and computational methods to support outcome prediction.

The authors of this study used a consecutive case series in which the following data were collected during presentation: age, sex, time from last seen well to arrival at hospital, NIHSS score on admission, laboratory tests and results of (TOAST) classification, history of smoking, hypertension, diabetes mellitus, atrial fibrillation, prior ischemic stroke, ischemic penumbra volume, and infarct core volume CNNs are specialized neural networks designed for processing image data by capturing spatial features through convolutional layers. They are widely used in image classification, object detection, and medical image analysis. All intravenous thrombolysis was administered strictly within guideline-recommended time windows, ensuring adherence to established protocols. The 24-hour criterion mentioned applies solely to registry inclusion for cases such as wake-up stroke and to imaging-based selection. This approach does not imply thrombolysis beyond the recommended therapeutic window.

Inclusion criteria were adult patients (≥18 years) with acute ischemic stroke involving the anterior circulation who were treated with intravenous thrombolysis and had complete multimodal CT imaging (non-contrast CT, CT angiography, and CT perfusion), baseline and follow-up NIHSS assessments, and available 90-day mRS (modified Rankin Scale) outcomes.

Eligible patients had anterior circulation ischemic stroke caused by either severe large-artery stenosis (≥70%) or complete arterial occlusion, as confirmed by CT angiography. Both conditions were included because they share an ischemic mechanism, exhibit comparable perfusion deficits on CT perfusion imaging, and are treated with the same intravenous thrombolytic strategy in routine clinical practice. Culprit vessel status (severe stenosis *vs*. complete occlusion) was therefore recorded and considered in subsequent analyses to account for potential pathophysiological heterogeneity.

Exclusion criteria included posterior circulation stroke, intracranial hemorrhage on baseline CT, contraindications to intravenous thrombolysis, incomplete clinical or imaging data, and treatment with endovascular thrombectomy in addition to intravenous thrombolysis. Intravenous thrombolysis was performed with alteplase at a standard dose of 0.9 mg/kg (maximum 90 mg), with 10% of the total dose administered as an initial bolus, followed by a continuous intravenous infusion of the remaining dose over 60 minutes, in accordance with current guideline recommendations.

A rise in the NIHSS score of at least 4 points within the first 3 days following symptom onset was defined as END [16, 17]. Cases described as significant neurological decline requiring emergency care were limited to patients with documented objective worsening consistent with NIHSS-based criteria, ensuring standardized outcome classification. NIHSS assessments were conducted at specific times, including admission and the first 3 days after thrombolysis. Early neurologic deterioration occurred when a patient's NIHSS score increased by 4 or more points within the first 72 hours of testing, relative to their initial score. Certified stroke neurologists who had no role in the imaging analysis and who remained unaware of CT imaging details performed NIHSS scoring. The two experienced neurologists reached a consensus decision when they encountered uncertainty, which helped maintain assessment consistency. Significant declines that required emergency care were also labelled as END. The LFO results were evaluated using the mRS at 90 days. A score of 0-2 was considered a good 90-day mRS outcome.

### 2.2 Imaging Analysis

Patients underwent multimodal CT examination (including plain CT, CTA, and CTP) if the neurologist considered an acute stroke in the differential diagnosis or if the individual in question was known to have been asymptomatic no more than 24 hours prior to admission. The imaging was performed on a GE Revolution CT scanner. RNNs are designed to handle sequential data by maintaining memory of previous inputs through feedback connections. They are useful for tasks involving time-series data, such as patient monitoring and medical signal analysis. The non-contrast CT was set on 120 kV and 320 mAs, with a 0.625 mm slice thickness. Contrast-enhanced CTP was performed at 80 kV and 150 mAs during injection of about 40 mL of iodinated contrast at 5 mL/s, followed by a 50 mL saline flush. Subsequently, another scan (CTA) was carried out with a similar contrast injection amount and speed.

CT perfusion and CT angiography images were post-processed using RAPID software (iSchemaView, Menlo Park, CA, USA). Infarct core was defined as regions with relative cerebral blood flow (rCBF) <30% of the contralateral hemisphere, ischemic penumbra as tissue with time-to-maximum (Tmax) >6 seconds, excluding the infarct core, and perfusion lesion as tissue with Tmax >6 seconds, with whole-brain coverage obtained for CT perfusion acquisition. Image quality was reviewed prior to analysis, and scans with severe motion artifacts or inadequate contrast bolus were excluded. The regional leptomeningeal collateral rLMC (regional leptomeningeal collateral score) was assessed on CTA maximum intensity projection images using the previously described 20-point scoring system, while ASPECTS (Alberta Stroke Program Early CT Score) was evaluated on non-contrast CT according to standard criteria. All imaging assessments were independently performed by two experienced neuroradiologists blinded to clinical data and outcomes, with good inter-rater agreement for rLMC and ASPECTS scoring, and discrepancies resolved by consensus.

Automated image-processing software was used to quantify ischemic core and penumbral volumes. Early ischemic changes were assessed using the Alberta Stroke Program Early CT Score (ASPECTS) on non-contrast CT and CTA, with automated ASPECTS tools improving detection accuracy and inter-reader agreement. In addition, advanced CT-based selection strategies, including CT perfusion, were used to support imaging-guided decision-making in patients presenting beyond the early time window [18, 19]. Collateral circulation was evaluated using the regional leptomeningeal collateral (rLMC) score, a 20-point ordinal scale in which higher scores indicate more robust collateralization. In addition to collateral grading, CT perfusion-derived parameters were used to assess tissue viability and were shown to provide prognostic value for functional outcome in acute ischemic stroke prior to intervention [20]. Large-vessel stenosis and occlusion were assessed on CT angiography (CTA). Vascular patency was graded using a modified Thrombolysis in Myocardial Infarction (TIMI) scale, where complete occlusion was defined as the absence of distal contrast opacification, and severe stenosis was defined as critical luminal narrowing with markedly reduced distal flow. Multimodal CT imaging, including CTA and CT perfusion parameters, was used to characterize patients and predict early outcomes [21, 22]. Senior neuroradiologists performed all the operations.

### 2.3 Statistical Analysis

Statistical analyses were performed using SPSS version 26.0. Data were presented as n (%), mean ± SD, or median (IQR), as appropriate. Multivariable analyses were conducted to identify factors associated with early neurologic deterioration and long-term functional outcome after adjustment for relevant confounders [23, 24]. Model 1 included demographics, comorbidities, and imaging characteristics as independent variables. Model 2 additionally incorporated stroke severity indicators and covariates from Model 1: ICH within 24 hours, culprit artery, vascular occlusion, and baseline NIHSS score. Baseline NIHSS at admission was used for covariate adjustment, whereas early neurologic deterioration was defined based on changes in NIHSS during the first 72 hours after thrombolysis, thereby minimizing the risk of circular reasoning. Culprit vessel status (severe stenosis versus complete occlusion) was recorded and included as a covariate in the multivariable analyses to account for potential pathophysiological heterogeneity. Nonlinear relationships were further analyzed using restricted cubic splines. ROC curve analysis determined the optimal clinical predictor threshold. The Events-Per-Variable (EPV) ratio in the final multivariable models was approximately 2.63, ensuring model stability. The inclusion of infarct core, perfusion lesion, and ischemic penumbra was driven by their clinical relevance, despite potential collinearity. This approach remains hypothesis-generating, acknowledging the complexities in these interrelated variables.

Given the limited number of early neurologic deterioration events, multivariable models were constructed with attention to events-per-variable to reduce the risk of overfitting. Variables included in the final models were selected based on clinical relevance and univariable associations. The effective events-per-variable ratio was maintained at an acceptable level by model reduction. Missing data were minimal and handled using complete-case analysis. Model discrimination was evaluated using the area under the receiver operating characteristic curve, while calibration was assessed by comparing predicted and observed outcomes. Internal validation was limited by sample size; therefore, the reported ROC-derived cutoff values should be interpreted as exploratory and require external validation in independent cohorts.

## 3 RESULTS

In total, 131 patients were enrolled, with 23 (17.5%) having wake-up stroke and 18 (13.7%) having unclear daytime stroke onset. Baseline characteristics and post-thrombolysis factors are shown in Table **1**. Males accounted for 67.9% (89) and females, 32.1% (42), with a median age of 69 years (IQR 56-75) and onset-to-imaging time of 196 minutes (IQR 126-268). Hemorrhagic transformation occurred within 24 hours in 12%, with END in 21 (16%) patients. At day 90, for index events surrounding the day, the median mRS was 2 (IQR 0-4), and a good outcome was observed in 81 (61.8%) patients, with an associated 90-day mortality of 9.9%.

From the univariate perspective, END was associated with higher NIHSS, larger infarct core, larger ischemic penumbra (63 *vs*. 21 mL; *P* = 0.01), and larger perfusion lesion volumes (135.5 *vs*. 28.5 mL; *P* = 0.001). Also, END was associated with higher rates of symptomatic ICH (15.8% *vs*. 0%; *P* = 0.003), lower ASPECTS (9 *vs*. 10; *P* = 0.018), and lower rLMC scores (11 *vs*. 19; *P* = 0.001) (Table **2**). Transformers use attention mechanisms to process data by focusing on important features without relying on sequence order. They are highly effective in handling large datasets and are increasingly applied in both image and healthcare data analysis. In Model 1, adjusting for demographic variables and comorbidities, all CT imaging parameters were associated with END (*P*<0.05). When further adjusting for stroke severity indicators, the rLMC, infarct core (OR = 1.05; 95% CI: 1.02-1.08; *P* = 0.001), perfusion lesion (OR = 1.02; 95% CI: 1.01-1.03; *P* = 0.002), and ischemic penumbra (OR = 1.01; 95% CI: 1.00-1.02; *P* = 0.018) became associated predictors of END (Table **3**). Among patients with END, 9.5% had a favourable and 90.5% had an unfavourable later mRS at 90 days, compared with 71.8% and 28.2% in patients without END. END predicted an adverse outcome by 90.5% sensitivity and 71.8% specificity.

Table **3** reports the relationship between variables and early neurologic decline following stroke, along with odds ratios (OD) and 95% CIs. Statistically significant entries are shown in bold (*P*<0.05). is a model that includes various imaging parameters, age, arterial hypertension, and atrial fibrillation. Model 2 consists of Model 1 + initial NIHSS score, responsible artery, vessel occlusion, stroke aetiology, and intracranial haemorrhage within 24 hours. rLMC CI, ASPECTS, OR,mRS, NIHSS, and ASPECTS.

In attempting to determine favorable 90-day mRS outcomes, significant differences were found between patients with favorable LFO and those with unfavorable outcomes.

Table **4** presents a summary of baseline clinical characteristics evaluated between patients who achieved unfavorable functional outcomes (mRS 3-6) and those who achieved favorable outcomes (mRS 0-2) after 90 days. Patients who experienced unfavorable outcomes had higher baseline NIHSS scores than those who achieved favorable outcomes, with a median score of 13 compared to 4 (*P*<0.001). The two groups showed no significant differences in age, sex, body mass index, smoking status, diabetes mellitus, atrial fibrillation, or prior stroke history. The study found that patients with poor outcomes had higher hypertension rates, but the difference did not reach statistical significance.

Variables are given as median (IQR), mean ± standard deviation, or n (%). rLMC refers to the regional leptomeningeal collateral score; ASPECTS, NIHSS, CT, ICH, sICH, ICA, MCA, IQR, OTT, and END.

A representative case demonstrated dysarthria and right-sided hemiparesis with an initial NIHSS score of 8 is shown in Fig. (**1**). Multimodal CT imaging post-processed using RAPID software showed an ASPECTS score of 9 on non-contrast CT, good collateral circulation on CTA with an rLMC score of 18, and CT perfusion findings of a 9 mL infarct core and a 99 mL perfusion lesion, resulting in an ischemic penumbra greater than 90 mL. Severe stenosis of the right middle cerebral artery was observed. The patient was treated with intravenous thrombolysis, did not experience early neurologic deterioration, had an NIHSS score of 4 on day 2, and achieved a favorable functional outcome (mRS 1) at 90 days.

Table **5** presents results from a multivariable binary logistic regression analysis identifying imaging traits predictive of a favorable long-term functional outcome (mRS 0-2 at 90 days). Unadjusted analyses showed that higher ASPECTS and rLMC scores, along with smaller infarct core, perfusion lesion, and ischemic penumbra volumes, were associated with favorable outcomes; these associations remained significant after adjustment for demographic and vascular risk factors (Model 1). In the fully adjusted model (Model 2), the rLMC score, infarct core, perfusion lesion, and ischemic penumbra volumes remained significantly associated with favorable outcomes, whereas ASPECTS was no longer statistically significant.

A mechanism with good functional status (mRS 0-2 at three calendar months) was performed. Afterwards, the ORs and 95% CIs for stroke were calculated. Bold and diverse imaging parameters include sex, arterial hypertension, onset to imaging, and atrial fibrillation. Intercranial haemorrhage within 24 hours (Yes/No), artery responsible, vascular obstruction, and stroke aetiology. CI stands for confidence interval; mRS, NIHSS, or ASPECTS, and rLMC. Additionally, due to the weak linear relationship among infarct core, ischemic penumbra, perfusion lesion, and prognosis, we further verified the analysis by performing logistic segmented regression with restricted cubic splines. The results showed that the relationships between the infarct core, ischemic penumbra, perfusion lesion, and LFO were dramatically different across different segmented volumes; the OR values increased compared to the previous analysis, with detailed data provided in Table **6**.

The diagnostic testing capabilities of CT imaging parameters are demonstrated through receiver operating characteristic curves that analyze their ability to forecast initial neurological decline and subsequent functional recovery (Fig. **2**). The most effective method for forecasting early neurological decline in panel A uses perfusion lesion volume as the main predictor, with infarct core volume, ischemic penumbra volume, and rLMC score as secondary predictors. The rLMC score in panel B demonstrates a better ability to predict positive long-term functional outcomes than perfusion lesion, infarct core, and ischemic penumbra volumes, which show lower area under the curve values.

## 4 DISCUSSIONS

Predictive potential of CT imaging for END symptomatic AIS receiving thrombolytic therapy. Our finding reveals two key observations. Firstly, our comprehensive analysis of 131 patients indicated a strong association between END and long-term functional outcomes. Specifically, CT imaging parameters, including infarct core volume, perfusion lesion volume, ischemic penumbra volume, and rLMC score, were independently associated with END and LFO. These associations should be interpreted cautiously, as this was a retrospective single-center study with a limited number of early neurologic deterioration events. The findings are therefore exploratory and intended to generate hypotheses rather than be definitive.

Secondly, CT imaging parameters showed variable predictive value across volume segments. Our findings support the role of multimodal CT imaging in predicting early neurologic deterioration and functional outcomes in thrombolysis-treated AIS patients [5, 25-27]. Unlike previous work, this study provides an integrated evaluation of multimodal CT imaging parameters in thrombolysed anterior circulation stroke, simultaneously assessing early neurologic deterioration and long-term functional outcome within the same treatment context. This represents an incremental contribution rather than a replacement of existing prediction models. One of the scant investigations that integrates clinical factors with detailed imaging parameters to elucidate their combined impact on END and LFO among patients receiving thrombolytic therapy for AIS resulting from anterior circulation large-artery stenosis or occlusion. Transfer learning involves using a pre-trained model on a new but related task to improve performance with limited data. This approach is especially valuable in medical imaging, where labeled data is often scarce. Our findings offer valuable insights into the predictive factors for END and LFO within the same stroke mechanism and treatment strategy for anterior circulation stroke. By integrating clinical factors and detailed imaging parameters, we aim to provide a more comprehensive END and LFO in this patient population. Although severe stenosis (≥70%) and complete occlusion were adjusted for in the analysis, biological heterogeneity between these conditions may still exist. A descriptive analysis of Early Neurologic Deterioration (END) across these groups could offer further insights. Additionally, a sensitivity analysis stratified by culprit vessel status would help to reinforce the robustness of the findings.

In the present study, most patients presented with relatively mild stroke severity and experienced longer onset-to-treatment times. This observation is consistent with prior prospective trials in basilar artery and large-vessel occlusion strokes, which reported similar patterns of lower baseline severity despite delayed presentation [16, 28, 29]. However, END still affected a sizable fraction of these patients, and its onset was strongly correlated with poor functional outcomes. This suggests that the significant clinical importance of END is not limited to anterior circulation strokes alone or to posterior circulation strokes.

Patients with END had higher NIHSS scores, larger infarct core and penumbral volumes, and lower ASPECTS and rLMC scores, consistent with prior studies linking larger infarct volumes to increased END risk and more severe ischemic injury [30, 31]. Collateral status assessed by rLMC score was independently associated with long-term outcomes, highlighting the prognostic importance of collateral circulation in AIS [32]. Adequate collateral flow supports penumbral perfusion and promotes tissue salvage and functional recovery [33]. Impaired collateral remodeling has been attributed to oxidative stress, endothelial dysfunction, and disrupted nitric oxide signaling [34].

Interestingly, the infarct core, ischemic penumbra, perfusion lesion, and long-term functional prognosis were correlated differently across the various segmented volumes. This finding underscores the complexities of the relationships between these imaging variables and clinical outcomes [10]. To better understand, these relationships were subjected to segmented regression analyses with restricted cubic splines, which showed that the associations between the imaging parameters and long-term functional prognosis differ across segmented volume conditions. Still, in the present sample, a higher proportion of patients with smaller infarct core volumes might have introduced bias, making it necessary in the future to very accurately and in detail elucidate the relationships between imaging parameters and prognosis.

ROC curve analysis revealed that a larger perfusion lesion volume is associated with END, whereas rLMC scores better predict long-term functional outcomes. These findings imply that the volume of perfusion lesions and the rLMC score might be used as imaging markers to predict END and long-term functional prognosis, respectively [3]. However, one point to consider is that the AUC values obtained in this study were moderate. In addition, the limited number of events restricted the complexity of multivariable modeling, and the reported predictive performance should be interpreted with caution due to the potential risk of overfitting. This suggests they have moderate predictive power; hence, other factors must be combined to improve it.

The retrospective single-center design, modest sample size, and limited number of early neurologic deterioration events represent important limitations of this study. The inclusion of both severe stenosis and complete occlusion may introduce biological heterogeneity; however, adjustment for culprit vessel status and imaging-based selection were used to mitigate this effect, although residual time-dependent bias cannot be fully excluded in this retrospective design. Infarct core, ischemic penumbra, and perfusion lesion volumes are intrinsically correlated, which may introduce collinearity in multivariable models; therefore, these variables were interpreted cautiously and analyzed in a hypothesis-generating manner. These factors constrained multivariable modeling and precluded internal or external validation. Missing data were minimal and handled by complete-case analysis. Future prospective multicenter studies with larger sample sizes and validation cohorts are required to confirm these findings and to establish robust imaging-based prediction models for early neurologic deterioration and functional outcome in thrombolysed acute ischemic stroke.

## CONCLUSION

This retrospective single-center study suggests that multimodal CT imaging parameters, including regional leptomeningeal collateral score and measures of ischemic tissue volumes, are associated with early neurologic deterioration and 90-day functional outcomes in thrombolysed patients with acute ischemic stroke. Self-supervised learning allows models to learn meaningful representations from unlabeled data by creating surrogate tasks. It reduces the need for manual annotation, making it useful for large-scale healthcare datasets. These parameters were analyzed to evaluate their potential role in predictive modeling of neurological outcomes. Given the limited number of early neurologic deterioration events and the absence of internal or external validation, these findings should be interpreted as exploratory. Further prospective, multicenter studies are required to confirm these associations and determine their clinical applicability. The ROC-derived thresholds identified in this study are exploratory and highlight discriminatory performance rather than predictive power. These thresholds should be interpreted with caution until externally validated in independent cohorts. Further validation is essential before clinical application.

## Figures and Tables

**Fig. (1):**
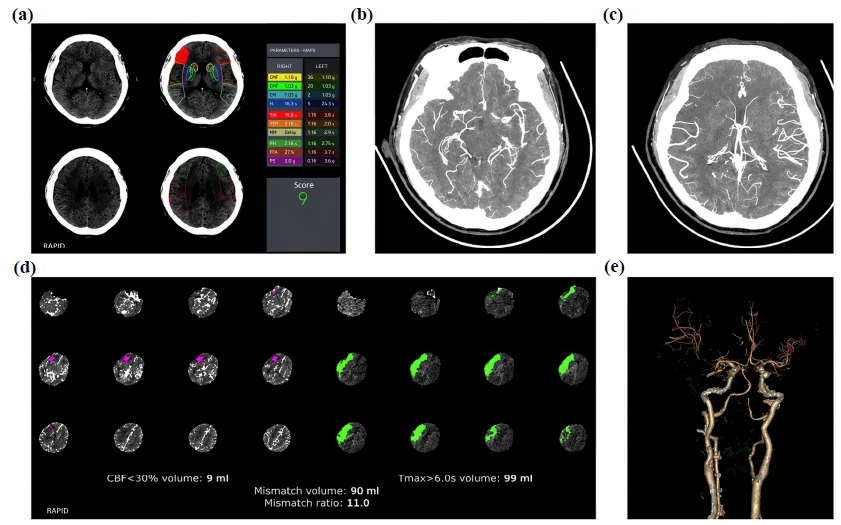
Pictures displaying dysarthria and hemiparesis on the right side, with an NIHSS scoring of 8 at onset, with an approximate time of 4 hours. The patient was immediately taken for a 1-stop CT scan, including CTA and CTP, and the scans were automatically post-processed using Rapid software. (**a**) ASPECTS scored 9. (**b**, **c**) The rLMC collateral circulation score was assigned 18 for this patient on CTA MIP imaging evaluation. (**d)** CTP images showed a 9 mL infarct core volume and a 99 mL perfusion lesion volume, with an ischemic penumbra of>90 mL. (**e**) CTA silhouette images revealed severe stenosis at the right MCA. The patient was treated with thrombolysis. On the second day post-stroke, the NIHSS score was reassessed as 4, without the occurrence of END. At 90 days, the patient's Modified Rankin Scale score was 1.

**Fig. (2):**
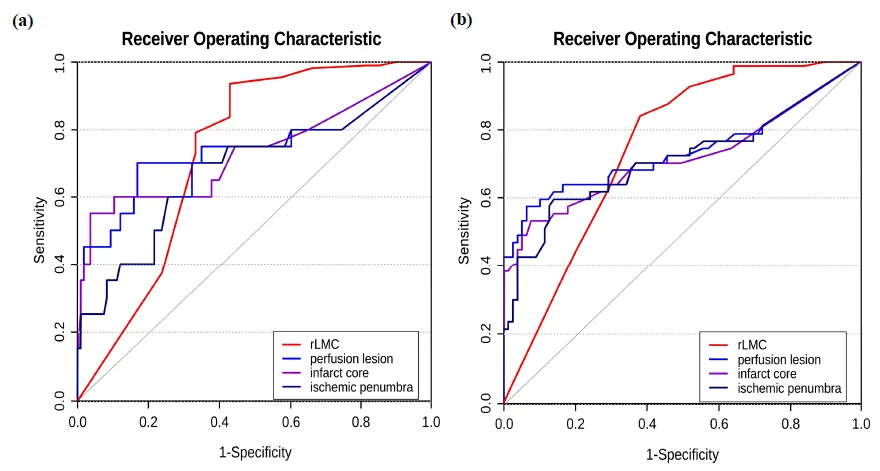
Receiver operator imaging parameter characteristic curves. (**a**) The perfusion lesion yielded the highest AUC (0.738) for END prediction. The highest AUC value for rLMC in predicting long-term functional prognosis was 0.751 (**b**). AUC is for area under the curve; rLMC stands for lower regional leptomeningeal collateral; and END stands for early neurological dysfunction.

**Table 1:** Demographic information combined with outcome and clinical profiles.

**Parameter**	**Value**
**Demographic and Clinical Profiles**	
Age, years	69 (56-75)
Sex, Male	89 (67.9)
BMI	24±3.5
Smoking	45 (34.4)
Hypertension	81 (61.8)
Diabetes mellitus	30 (22.9)
Atrial fibrillation	44 (33.6)
Previous stroke	14 (10.7)
Initial NIHSS score	7 [3-13]
Onset to imaging, min	196 (126-268)
OTT, min	240 (167.5-370)
**Imaging or Angiography Profiles**	
Responsible artery	
ICA	27 (20.6)
MCA	98 (74.8)
ACA	6 (4.6)
Occlusion	73 (55.7)
**Stroke Etiology**	
Large-artery atherosclerosis	78 (59.5)
Cardioembolism	35 (26.7)
Small-artery occlusion	11 (8.4)
Other etiology	4 (3.1)
Undetermined etiology	3 (2.3)
ASPECTS score	10 [9-10]
rLMC score	19 [16-20]
Infarct core, mL	2 (0-14)
Perfusion lesion, mL	37 (5-91)
Ischemic penumbra, mL	30.5 (3.2-66.8)
**Outcome Profiles**	
ICH within 24 hours	15 (11.5)
Symptomatic ICH	3 (2.4)
END	21 (16.0)
mRS at 90 days	2 (0-4)
Favourable mRS score at 90 days	81 (61.8)
Mortality at 3 months	13 (9.9)

**Note:** Continuous variables are given as median (IQR) or n (%). NIHSS, ASPECTS, HI, ICH, TICI, IQR; Mrs, END, OTT).

**Table 2:** Factors associated with END.

**Parameter**	**No END (n=110)**	**END (n=21)**	***P*-value**
Age, years	70 (57-76)	62 (48-70)	0.139
Sex, Male	75 (68.2)	14 (66.7)	0.892
BMI, kg/m^2^	23.8±3.5	24.7±3.5	0.286
Smoking	37 (33.6)	8 (38.1)	0.693
Hypertension	67 (60.9)	14 (66.7)	0.619
Diabetes mellitus	25 (22.7)	5 (23.8)	1.000
Atrial fibrillation	35 (31.8)	9 (42.9)	0.326
Previous stroke	11 (10.0)	3 (14.3)	0.844
Initial NIHSS score	7 (3-11)	12 (4-18)	<0.001
Onset to imaging, min	193.5 (126-272.2)	198 (154-243)	0.913
OTT, min	237.5 (170-373.8)	245 (139-290)	0.851
Responsible vessel			0.004
Occlusion	59 (53.6)	14 (66.7)	0.271
Stroke etiology			0.199
ASPECTS score	10 (9-10)	9 (7-10)	0.018
rLMC score	19 (17-20)	11 (9-19.2)	0.001
Infarct core, mL	2 (0-10)	84 (2.5-140.8)	0.001
Perfusion lesion, mL	28.5 (1.25-67)	135.5 (40.25-288.25)	0.001
Ischemic penumbra, mL	21 (0.8-57.5)	63 (28-122.8)	0.01
ICH within 24 hours	12 (11.3)	3 (15.8)	0.866
sICH	0 (0)	3 (15.8)	0.003
mRS at 90 days	1 (0-3)	5 (3-6)	<0.001

**Note:** Variables are represented by median with corresponding IQR, mean ± standard deviation, or number (percentage). END: early neurologic deterioration; (ASPECTS); (rLMC (NIHSS); (CT); (MCA (ICA); (ICH); (sICH); IQR: OTT.

**Table 3:** Analysis of multivariable binary logistic regression.

**Variable**	**Unadjusted OR**	***P*-value**	**Model 1**	***P*-value**	**Model 2**	***P*-value**
ASPECTS	0.67 (0.51-0.88)	0.005	0.68 (0.50-0.91)	0.011	0.74 (0.49-1.10)	0.131
rLMC score	0.76 (0.67-0.85)	0.001	0.71 (0.61-0.83)	0.001	0.61 (0.47-0.81)	0.001
Infarct core	1.02 (1.01-1.03)	0.001	1.03 (1.01-1.04)	0.001	1.05 (1.02-1.08)	0.001
Perfusion lesion	1.01 (1.06-1.02)*	0.001	1.01 (1.01-1.02)	0.001	1.02 (1.01-1.03)	0.002
Ischemic penumbra	1.01 (1.06-1.02)*	0.001	1.01 (1.01-1.02)	0.001	1.01 (1.00-1.02)	0.018

**Table 4:** Factors associated with clinical functional outcomes.

**Parameter**	**mRS 3-6 (n=50)**	**mRS 0-2 (n=81)**	***P*-value**
Age, years	70 (57-79)	68 (55-74)	0.123
Sex, Male	33 (66.0)	56 (69.1)	0.709
BMI	24.5±3.7	23.7±3.3	0.227
Smoking	19 (38.0)	26 (32.1)	0.49
Hypertension	36 (72.0)	45 (55.6)	0.06
Diabetes mellitus	14 (28.0)	16 (19.8)	0.275
Atrial fibrillation	19 (38.0)	25 (30.9)	0.401
Previous stroke	6 (12.0)	8 (9.9)	0.702
Initial NIHSS score	13 (9-16.8)	4 (2-8)	**<**0.001

**Table 5:** Analysis of MBLR*.

**Variable**	**Unadjusted OR**	***P***	**Model 1**	***P*-value**	**Model 2**	***P*-value**
**ASPECTS**	1.80 (1.29-2.5)	**0.001**	1.79 (1.29-2.48)	**0.001**	1.26 (0.87-1.84)	0.225
**rLMC score**	1.38 (1.20-1.60)	**0.001**	1.39 (1.20-1.60)	**0.001**	1.32 (1.09-1.59)	**0.004**
**Infarct core**	0.94 (0.91-0.97)	**0.001**	0.94 (0.91-0.97)	**0.001**	0.95 (0.91-0.99)	**0.007**
**Perfusion lesion**	0.98 (0.97-0.99)	**0.001**	0.98 (0.97-0.99)	**0.001**	0.99 (0.98-1.00)	**0.005**
**Ischemic penumbra**	0.98 (0.97-0.99)	**0.001**	0.98 (0.97-0.99)	**0.001**	0.99 (0.97-1.00)	**0.024**

**Table 6:** Logistic segmented regression analysis *via* restricted cubic splines^†^.

**Segmented Variable**	**Segmented Points**	**Slope**	***t***	***P*-value**	**OR**	**95% CI**
Infarct core	2.0	Slope 1	1.053	0.292	3.503	0.339-36.145
		Slope 2	−2.528	**0.011**	**0.908**	**0.842**-**0.978**
Ischemic penumbra	16.0	Slope 1	2.450	**0.014**	**1.335**	**1.059**-**1.681**
		Slope 2	−3.205	**0.001**	**0.960**	**0.937**-**0.984**
Perfusion lesion	25.9	Slope 1	2.324	**0.020**	**1.223**	**1.032**-**1.449**
		Slope 2	−3.584	**0.001**	**0.955**	**0.932**-**0.98**

**Note:** ^†^, Logistic regression analysis of max-sis according to the connection for the pooled cohort. An mRS of 0-2 was predicted by the interaction between ischemic penumbra and infarct core three months after stroke; odds and 95% CI.

## Data Availability

The datasets generated during and/or analyzed during the current study are available from the corresponding authors [S.Z] and [X.Y] on reasonable request and are included within this published article.

## LIST OF ABBREVIATIONS

AIS: Acute Ischemic Stroke
END: Early Neurologic Deterioration
CT: Computed Tomography
CTA: CT Angiography
CTP: CT Perfusion
NCCT: Non-Contrast Computed Tomography
ASPECTS: Alberta Stroke Program Early CT Score
rLMC: Regional Leptomeningeal Collateral Score
NIHSS: National Institutes of Health Stroke Scale
mRS: Modified Rankin Scale
ICH: Intracerebral Hemorrhage
sICH: Symptomatic Intracerebral Hemorrhage
OTT: Onset-to-Treatment Time
ROC: Receiver Operating Characteristic
AUC: Area Under the Curve
OR: Odds Ratio
CI: Confidence Interval
TOAST: Trial of ORG 10172 in Acute Stroke Treatment
ICA: Internal Carotid Artery
MCA: Middle Cerebral Artery
ACA: Anterior Cerebral Artery
TIMI: Thrombolysis in Myocardial Infarction
SD: Standard Deviation
IQR: Interquartile Range

## STANDARDS OF REPORTING

STROBE guidelines were followed.
